# Butyrylcholinesterase Genetic Variants: Association with Cocaine Dependence and Related Phenotypes

**DOI:** 10.1371/journal.pone.0080505

**Published:** 2013-11-27

**Authors:** André Brooking Negrão, Alexandre Costa Pereira, Camila Guindalini, Hadassa Campos Santos, Guilherme Peres Messas, Ronaldo Laranjeira, Homero Vallada

**Affiliations:** 1 Department and Institute of Psychiatry (LIM 23), University of São Paulo Medical School, São Paulo, SP, Brazil; 2 Laboratory of Genetics and Molecular Cardiology (LIM 13), Heart Institute-InCor, University of São Paulo Medical School, São Paulo, SP, Brazil; 3 Department of Psychobiology, Universidade Federal de São Paulo, São Paulo, Brazil; 4 Clinical Neuroscience Laboratory, Department of Psychiatry, Universidade Federal de São Paulo, São Paulo, SP, Brazil; 5 School of Public Health, University of São Paulo, São Paulo, SP, Brazil; 6 National Institute of Alcohol and Drug Policies, Universidade Federal de São Paulo, São Paulo, SP, Brazil; Penn State College of Medicine, United States of America

## Abstract

**Objective:**

The search for genetic vulnerability factors in cocaine dependence has focused on the role that neuroplasticity plays in addiction. However, like many other drugs, the ability of an individual to metabolize cocaine can also influence susceptibility to dependence. Butyrylcholinesterase (BChE) metabolizes cocaine, and genetic variants of the BChE gene (BCHE) alter its catalytic activity. Therefore, we hypothesize that cocaine users with polymorphisms in BCHE can show diverse addictive behaviors due to differences in effective plasma concentrations of cocaine. Those polymorphisms might also influence users to prefer one of the two main preparations (crack or powder cocaine), despite having equal access to both. The present work investigates polymorphisms in BCHE and if those genetic variants constitute risk factors for cocaine dependence and for crack cocaine use.

**Methods:**

A total of 1,436 individuals (698 cocaine-dependent patients and 738 controls) were genotyped for three single nucleotide polymorphisms (SNPs) in BCHE: rs1803274, rs4263329, and rs4680662.

**Results:**

For rs4263329, a nominal difference was found between cases and controls. For rs1803274 (the functional SNP), a statistically significant difference was found between patients who used crack cocaine exclusively and those who used only powder cocaine (P = 0.027; OR = 4.36; 95% CI = 1.18–16.04). Allele frequencies and genotypes related to other markers did not differ between cases and controls or between the two cocaine subgroups.

**Conclusions:**

Our findings suggest that the AA genotype of rs1803274 is a risk factor for crack cocaine use, which is more addictive than powder cocaine use. Further studies are needed in order to confirm this preliminary result and clarify the role of BCHE and its variants in cocaine dependence.

## Introduction

Cocaine addiction is a complex behaviour that arises from the interactions between genetic and environmental risk factors. Twin studies indicate that the heritability of cocaine addiction is approximately 60% and fits a complex polygenic model [1]. Cocaine binds strongly to the dopamine transporter, and this blockade of dopamine reuptake is perhaps the key mechanism leading to cocaine addiction [2]. In fact, some studies have already reported genetic markers in dopaminergic brain systems associated with cocaine dependence [3]–[5]. However, many of those markers are also associated with other psychiatric disorders or substance-related disorders other than cocaine-related disorders and therefore might not be specific markers for the cocaine dependency risk [6]–[8]. The results of twin studies indicate that the heritability of substance dependence can be general or drug-specific, suggesting that specific genetic markers increase susceptibility to dependence on distinct drugs [9], [10]. In one of the largest studies of cocaine dependence to date [3] various polymorphisms were found to be associated with the cocaine-dependent phenotype, as has also been demonstrated in animal studies involving markers related to the dopaminergic reward system and to other biochemical pathways [11], [12].

Although cocaine users use the drug in various forms, most can be distinguished by their preferred route of cocaine administration: inhalation (smoking crack cocaine); nasal (snorting powder cocaine); or injection (injecting a cocaine preparation) [13]. Those subgroups have been associated with specific characteristics of cocaine use: escalation of consumption; degree of abuse liability; propensity for dependence; and treatment response [14], [15]. It is argued that the reinforcing effect of smoked (crack) cocaine is greater than is that of snorted (powder) cocaine because, when the drug is smoked, the peak effect is achieved more quickly and concentrations of the drug in the central nervous system are higher for an equivalent amount of cocaine consumed [16]. Nevertheless, most cocaine-dependent patients have a preferred route of administration, despite having been exposed to both forms [13]. Therefore, the preferred route of administration can represent a distinct phenotype among cocaine users and should be taken into account when investigating individual genetic susceptibility to cocaine use and abuse.

One limitation of previous studies on genetic susceptibility to cocaine dependence, as well as to other complex disorders, is that known polymorphisms may explain only a small fraction of the heritability variance in cocaine dependence. Therefore, there is a need for studies investigating putative additional genetic markers associated with susceptibility to cocaine dependence, in particular those that might be specific to this disorder [17].

Pharmacogenetic factors, especially genetic aspects that modulate the plasma concentration of cocaine, could play a role in cocaine susceptibility and have yet to be studied. Once absorbed, cocaine is rapidly transformed in two main metabolites, benzoylecgonine and ecgonine methyl ester, both of which are pharmacologically inactive [18]. The hydrolysis that leads to the formation of ecgonine methyl ester is catalyzed by butyrylcholinesterase (BChE), which is an enzyme involved in the metabolism of certain drugs (including cocaine and heroin), various local anesthetics, and short-acting muscle relaxants [19]. BChE is synthesized primarily in the liver and is distributed throughout the intestinal mucosa, in plasma, and in the white matter of the central nervous system [20]. The enzyme is encoded by the BChE gene (BCHE), which is located on chromosome 3q26 [21]. The BCHE genomic region spans approximately 70 kb, with four exons and three large introns [22]. Although more than 65 BCHE mutations have been identified, not all of them have been fully studied [23]. In general, these mutations produce enzymes with lower levels of catalytic activity than that of those produced by wild-type mutations [24]. BChE has also been tested as a novel therapeutic agent for cocaine dependence: a quadruple mutant hydrolase derived from human BChE suppressed cocaine toxicity and abolished drug-primed reinstatement in rats [25]. Our working hypothesis is that polymorphisms in BCHE lead to various enzyme profiles that allow different concentrations of cocaine to reach the reward system in the brain, thereby increasing or decreasing susceptibility to developing addictive behaviors.

The objective of this study is to identify genetic variations in BCHE as risk factors for dependence in patients whose primary drug of abuse is cocaine. We also investigate those genetic markers and the preferred route of cocaine administration for any existing correlation.

## Methods

The patient sample consisted of 698 cocaine-dependent patients [mean age 26.8±7.2 years; 96% males (n = 669)], recruited and evaluated as inpatients and outpatients from seven drug dependence treatment clinics in São Paulo, Brazil. All of the patients met the criteria for cocaine dependence established in the tenth revision of the International Classification of Diseases. At enrollment, each patient was subjected to a screening interview, designed specifically for use in Brazil, that included questions related to sociodemographic variables and to drug use [26].

We recruited 738 unrelated controls [mean age 31.3±9.8 years and 68% males (n = 501)] from the Fundação Pró-Sangue, Hospital das Clínicas, Universidade de São Paulo (Blood Donation Center at the University of São Paulo School of Medicine). Exclusion criteria were: those with a history of drug abuse or recent use of illicit drugs, as were those with a history of psychiatric inpatient treatment or with a current psychiatric condition.

To conduct a comparative analysis according to the route of cocaine administration, we divided the patient sample into three subgroups: those who reported using only powder cocaine (typically by snorting); those who reported using only freebase cocaine hydrochloride (crack cocaine) which is smoked in small pipes and, those who reported using both routes of cocaine administration (dual users). A detailed description of the above subgroups can be found elsewhere [13].

### Ethics Statement

The study was approved by the Comissão de Ética para a Análise de Projeto de Pesquisa (CAPPesq, Ethics Committee for the Analysis of Research Projects) of the University of São Paulo School of Medicine. All participants provided written informed consent.

### Genotyping

All of the participants were genotyped for three single nucleotide polymorphisms (SNPs) in BCHE: rs1803274, rs4263329, and rs4680662. To that end, blood samples were collected in tubes containing ethylenediaminetetraacetic acid, and genomic DNA was extracted by standard methods.

The SNP rs1803274 was selected because it is a common variant, known as the K variant, that leads to a functional decrease in BChE activity [24]. The other two SNPs were selected on the basis of the linkage disequilibrium (LD) structure of the gene, allele frequency, and available Haplotype Map data (http://hapmap.ncbi.nlm.nih.gov/index.html.en). Genotyping was conducted by Prevention Genetics (Marshfield, Wisconsin, USA; http://www.preventiongenetics.com/). For all genotypes, the Hardy-Weinberg equilibrium was assessed using the Haploview software, version 4.2 [27]. To estimate the statistical power of the sample, we used the QUANTO program, version 1.2, assuming an odds ratio (OR) of 1.5, a disease prevalence of 0.03, a calculated average minor allele frequency of 0.27, and a significance level of 0.05 [28].

**Figure 1:**
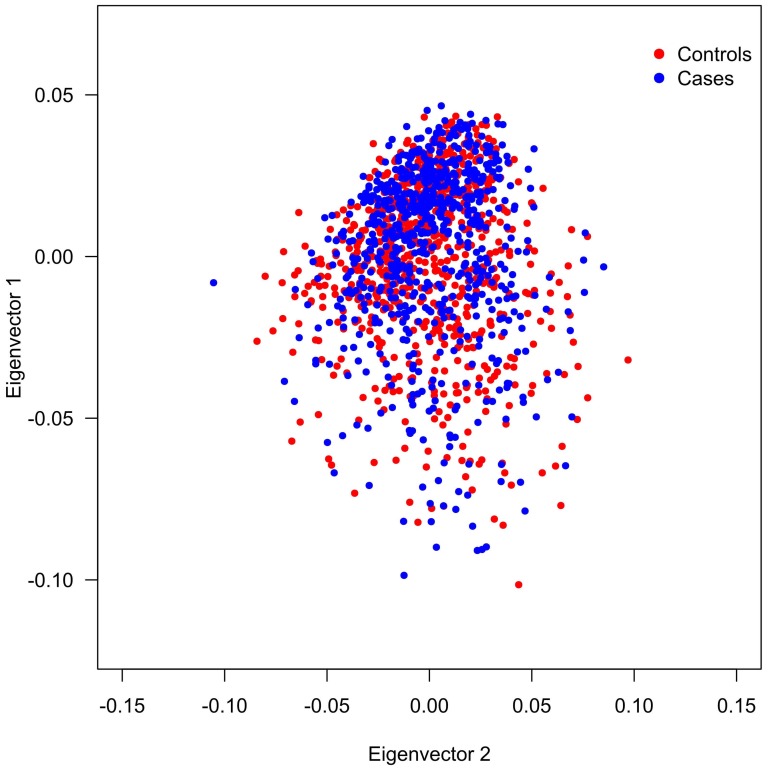
Population Structure Analysis. Graphic representation of the first two principal components for cases and controls genotyped with 64 AIMs, each point in this plot is an individual. The distribution of individuals in the axes is similar for both groups therefore the EIGENSTRAT software was not able to detect a difference in population stratification between cases and controls.

We performed principal component analysis (PCA) to assess allele frequency differences between cases and controls due to ancestry differences and statistical analyses were performed using EIGENSTRAT [29], [30]. This software detects population structure inferring axes of genetic variation and outputs each individual's coordinates along axes of variation. The analysis was performed using a panel of 64 SNPs ancestry-informative markers (AIMs) and models were created with two to five principal components in order to detect the existence or absence of population structure; details of marker set are available on request. In our data set, we were not able to demonstrate a difference in population stratification between cases and controls therefore, no corrections using the PCA results were done in the association tests for the BCHE markers (Figure 1).

A general test of association (the two-degree-of-freedom test of genotypic association) was applied, and three individual contrasts, defined by a priori genetic models (additive, dominant, and recessive), were computed. In the dominant and recessive models, explanatory variables were binary. We employed logistic regression to adjust for age and sex. No statistical correction was made for multiple comparisons in the general test of association. The LD and the haplotype frequencies were estimated with the Haploview software, version 4.2. Haplotype blocks were identified using the solid spine of LD method in Haploview and correction for multiple testing was performed using permutation correction by the Haploview program [27]. Chi-square values, ORs, and 95% confidence intervals (95% CIs) were estimated using the Statistical Package for the Social Sciences, version 17.0 (SPSS Inc., Chicago, Illinois, USA).

## Results

The socio-demographic and clinical characteristics of the participants are summarized in Table 1. The patient and control groups differed significantly in terms of sex and age. Most of the patients used cannabis and were cigarette smokers. Roughly half of the patients ingested more than 50 units of alcohol per week and had been in prison at least once. In terms of the preferred route of cocaine administration, 23% of the participants reported using the nasal route (snorting powder cocaine) exclusively, 9% reported using the inhalation route (smoking crack cocaine) exclusively, and 68% reported using both routes concurrently (dual users). For cases and controls, none of the allele distributions deviated significantly from those expected on the basis of the Hardy-Weinberg equilibrium (Table 2). When we adopted a recessive model of transmission, the GG genotype of rs4263329 was less common in cases than in controls [f(GG) = 1.2% vs. f(GG) = 2.6%; OR 2.3, 95% CI = 0.99–5.32]. After adjustment for age and sex, that association was no longer significant (P>0.1). As can be seen in Table 2, genotypes of the two other markers did not differ between cases and controls, nor did the allele frequencies of the three markers. Measures of LD and haplotype blocks across BCHE did not produce any evidence for an association with disease (data not shown). Results of the general tests of association for the preferred route of cocaine administration can be seen in Table 3. Of the three markers, only rs1803274 was associated with distinct genotypes among the subgroups of cocaine users. Among the a priori genetic models, the recessive model (genotype AA) best accounted for the significant difference between crack users and powder cocaine users (P = 0.027; OR = 4.36; 95% CI = 1.18–16.04), as well as for that observed between crack users and dual users (P = 0.001; OR = 5.83; 95% CI = 2.10–16.16). Those associations remained significant after adjustment for age and sex (data not shown).

**Table 1:** Characteristics of cocaine-dependent patients and control subjects.

Characteristic	Patients	Controls
	(n = 698)	(n = 738)
Age, in years, mean ± SD (range)	26.8±7.2 (17–56)	31.3±9.8 (18–72)
Sex		
Male, n (%)	669 (95.8)	501 (67.9)
Female, n (%)	29 (4.2)	237 (32.1)
Preferred route of administration		
Nasal (powder cocaine users), n (%)	160 (22.9)	
Inhalation (crack cocaine users), n (%)	61 (8.7)	
Either (dual users), n (%)	477 (68.3)	
Cigarette smoking		
Yes, n (%)	585 (83.8)	
No, n (%)	111 (15.9)	
Alcohol consumption		
<50 units/week, n (%)	417 (59.7)	
>50 units/week, n (%)	247 (35.4)	
None, n (%)	34 (4.8)	
History of cannabis use		
Yes, n (%)	666 (95.4)	
No, n (%)	23 (3.3)	
No data, n (%)	9 (1.3)	
Criminal history		
Yes, n (%)	373 (53.4)	
No, n (%)	324 (46.4)	
No data, n (%)	1 (0.1)	

SD, standard deviation.

**Table 2:** Genotype and allele frequencies of three BCHE markers in cocaine-dependent patients and controls.

	Genotype				Allele		HWE (P)
SNP/Group	AA	AG	GG	n	G	A	
rs1803274							
Patients, n (%)	18 (2.7)	201 (29.7)	457 (67.6)	676	0.82	0.18	1.0
Controls, n (%)	30 (4.2)	215 (29.8)	477 (66.1)	722			
rs4263329^a^							
Patients, n (%)	497 (72.6)	180 (26.3)	8 (1.2)	685	0.14	0.86	1.0
Controls, n (%)	538 (74.5)	165 (22.9)	19 (2.6)	722			
rs4680662							
Patients, n (%)	75 (10.9)	317 (46.0)	297 (43.1)	689	0.66	0.34	0.71
Controls, n (%)	81 (11.6)	316 (45.1)	303 (43.3)	700			

SNP, single nucleotide polymorphism; HWE, Hardy-Weinberg equilibrium.

a rs4263329 GG genotype, P = 0.052, odds ratio = 2.3 (95% confidence interval = 0.99–5.32).

**Table 3:** Genotype frequencies of three BCHE markers in cocaine-dependent patients, by preferred form of cocaine administration.

	Genotype			n	χ^2^	P
SNP/Subgroup	AA	AG	GG			
rs1803274^a^						
Powder cocaine users, n (%)	4 (2.6)	45 (29.0)	106 (68.4)	155		
Crack users, n (%)	6 (10.6)	14 (24.1)	38 (65.5)	58		
Dual users, n (%)	8 (1.7)	142 (30.7)	313 (67.6)	463		
Total, n (%)	18 (2.7)	201 (29.7)	457 (67.6)	676	15.20	0.004
rs4680662						
Powder cocaine users, n (%)	16 (10.1)	77 (48.7)	65 (41.1)	158		
Crack users, n (%)	4 (6.6)	29 (47.5)	28 (45.9)	61		
Dual users, n (%)	55 (11.7)	211 (44.9)	204 (43.4)	470		
Total, n (%)	75 (10.9)	317 (46.0)	297 (43.1)	689	2.10	0.718
rs4263329						
Powder cocaine users, n (%)	107 (68.2)	46 (29.3)	4 (2.5)	157		
Crack users, n (%)	46 (76.7)	13 (21.7)	1 (1.7)	60		
Dual users, n (%)	344 (73.5)	121 (25.9)	3 (0.6)	468		
Total, n (%)	497 (72.6)	180 (26.3)	8 (1.2)	685	5.48	0.241

a rs1803274 AA genotype, P = 0.027; OR = 4.36 (95% confidence interval = 1.18–16.04), between crack users and powder users.

## Discussion

We evaluated the distribution of three SNPs in BCHE in a sample of cocaine-dependent patients and controls in Brazil. For the SNP rs4263329, there was a nominal association between patients and controls [GG genotype, P = 0.05, OR = 2.3; 95% CI = 0.99–5.32]. We also found a significant association between rs1803274 and crack cocaine (AA genotype) as the preferred route of administration (p<0,005). To our knowledge, this is the only report in the literature investigating BCHE variants and crack/cocaine.

We are aware that these results could be false positive ones, but in order to minimize the likelihood of it, we used the best available methodology and analysis. Firstly, the investigated sample is one of the largest samples of crack/cocaine patients reported in the literature to study genetic susceptibility for cocaine dependence to date. We also adjusted for confounding factors (age and sex) to confirm that the association was not due to bias. Population stratification was tested with the EIGENSTRAT software and no differences were observed between cases and controls which could hinder the association results. In contrast with other studies investigating the association of genetic markers and cocaine dependence [31], [32], subjects in the present study were stratified by their preferred route of drug administration. This may decrease the sample's clinical heterogeneity, which frequently reduces the power of genetic association studies. Another confounding factor in previous studies was a concomitant dependence on other drugs, mainly alcohol and heroin. In the present study, individuals with alcohol dependence were excluded, and heroin dependence is very rare in Brazil (less than 1% of our sample used heroin). This attempt to make the sample more homogeneous may strengthen the specificity of any genetic association found as related to the condition of cocaine use per se. From a statistical standpoint, our sample had a power of 88% of detecting true associations.

One of the markers investigated in our study, rs1803274, was not associated with cocaine dependence but was associated with crack smoking as the preferred route of administration. Although the rs1803274 marker has previously been shown to be associated with behavioral and medical conditions [33], [34], we found no studies investigating the role of rs1803274 in substance dependence or other substance-related psychiatric disorders to date. The fact that we found an association only when the sample was divided into subgroups is not unique. An association with a genetic marker in cocaine dependence was found when the sample was subdivided into those who had experienced psychotic symptoms during episodes of cocaine intoxication and those who had not [35]. Because cocaine dependent subjects constitute a heterogeneous group not only from a genetic standpoint but also from a clinical perspective, it is useful to study them in meaningful subgroups. Dual users have distinct clinical features that distinguish them from those who are exclusive users of crack or snorted cocaine [13]. In the present study, allele frequencies for rs1803274 were very similar to the values for the Haplotype Map population of Utah residents with ancestry from northern and western Europe (CEU population). We also found that the frequency of the AA genotype in our sample as a whole was greater than in the CEU population (3.3% compared to 1.7%). The other two markers investigated have been infrequently used in association studies. Twelve BCHE SNPs were tested in farmers exposed to organophosphates and rs4680663 was not found to be associated with cholinesterase activity [36]. We found no association studies using the SNP rs4263329 as a marker.

Common variants have been associated with altered cholinesterase activity in samples drawn from the community [36]. The SNP rs1803274 is a common variant in BCHE, and the A allele leads to a point mutation at nucleotide 1615 that changes codon 539 from GCA (ala) to ACA (thr) [38]. In carriers of the A allele, serum BChE levels are reduced by 30%. Decreased BChE activity increase the amount of cocaine that reaches the reinforcing brain areas, thereby augmenting its propensity to lead to dependence. Most cocaine users in Brazil have equal access to crack and powder cocaine, and the AA genotype can, in part, explain the fact that some users prefer to use crack cocaine exclusively. It is likely that the enzymatic changes interact with other known predisposing factors for crack dependence and account for part of the genetic vulnerability to this route of administration.

Like any genetic association study, our study has limitations that must be addressed. First, although we found a nominal association between rs4263329 and cocaine dependence, that association lost its significance after being adjusted for sex and age. Nevertheless, that is an interesting finding that warrants further studies in comparable populations. Second, our sample size, although large for this particular line of research, could be considered small for a genetic association study looking for a susceptibility gene with a small magnitude effect. Third, although the marker reported here to be associated with exclusive crack cocaine use has been correlated with functional enzymatic alterations in other studies, we did not obtain any cholinesterase measures, which would have allowed us to make a functional correlation between carrying the marker and its function in patients and controls [23], [36]. By the same token, we had no objective parameters of cocaine use in cases and controls, such as toxicological measurements of cocaine in hair or urine. The subjective ratings reported by cocaine users are known to be unreliable markers of the true amount and frequency of illegal substance use, and that holds true for self-reported drug use by non-dependent subjects, such as our controls [37]. Although we consider it unlikely, it is possible that our controls underreported cocaine use and dependence, which would have decreased the strength of the association found in the present study. Finally, it is possible that the BCHE SNP that is associated with vulnerability to crack use is not the actual causative SNP; instead, other nearby SNPs in LD could be the alternative causation.

Although some investigators have identified genetic components of susceptibility to cocaine dependence, only a small portion of the heritability is explained by those findings [17]. So many of the genetic susceptibility makers or genetic mechanisms for crack/cocaine dependence are still unknown. The identification of these new genetic markers will contribute to prevent and to treat drug abuse/dependent patients. In the present study, we evaluated three SNPs in BCHE, all of which are potentially involved in cocaine metabolism. Although we did not find an association between those markers and cocaine dependence per se, we observed an association between the known functional genotype (the K variant) and a preference for the inhalation (crack smoking) route of cocaine administration. Further studies involving a replication in other independent case-control samples and/or investigation involving a correlation between the function and the genetic variants and/or sequencing of this region would be very welcome to clarify the preliminary findings of the present report.
